# Modeling, Assessment, and Design of Porous Cells Based on Schwartz Primitive Surface for Bone Scaffolds

**DOI:** 10.1155/2019/7060847

**Published:** 2019-06-27

**Authors:** Rita Ambu, Anna Eva Morabito

**Affiliations:** ^1^Department of Mechanical, Chemical and Materials Engineering, University of Cagliari, Via Marengo 2, 09123 Cagliari, Italy; ^2^Department of Engineering for Innovation, University of Salento, Via per Monteroni, 73100 Lecce, Italy

## Abstract

The design of bone scaffolds for tissue regeneration is a topic of great interest, which involves different issues related to geometry of architectures, mechanical behavior, and biological requirements, whose optimal combination determines the success of an implant. Additive manufacturing (AM) has widened the capability to produce structures with complex geometries, which should potentially satisfy the different requirements. These architectures can be obtained by means of refined methods and have to be assessed in terms of geometrical and mechanical properties. In this paper a triply periodic minimal surface (TPMS), the Schwarz's Primitive surface (P-surface), has been considered as scaffold unit cell and conveniently parameterized in order to investigate the effect of modulation of analytical parameters on the P-cell geometry and on its properties. Several are the cell properties, which can affect the scaffold performance. Due to the important biofunctional role that the surface curvature plays in mechanisms of cellular proliferation and differentiation, in this paper, in addition to properties considering the cell geometry in its whole (such as volume fraction or pore size), new properties were proposed. These properties involve, particularly, the evaluation of local geometrical-differential properties of the P-surface. The results of this P-cell comprehensive characterization are very useful for the design of customized bone scaffolds able to satisfy both biological and mechanical requirements. A numerical structural evaluation, by means of finite element method (FEM), was performed in order to assess the stiffness of solid P-cells as a function of the changes of the analytical parameters of outer surface and the thickness of cell. Finally, the relationship between stiffness and porosity has been analyzed, given the relevance that this property has for bone scaffolds design.

## 1. Introduction

The interest in the development of three-dimensional structures, generally termed as bone scaffolds, to be used as bone substitutes has grown over time [1]. The enhanced capability of new manufacturing methods, such as Additive Manufacturing (AM) [2, 3] has encouraged the design of structures with more complex architectures to better satisfy the necessary requirements for this kind of application.

Generally, porous structures with interconnected pores are required with a geometrical configuration, for example, for promotion of cell ingrowth and transport of nutrients. Consequently, proper methodologies of modeling have to be considered to obtain these structures. Different approaches have been proposed for their design. These comprise CAD-based methods [4, 5], image-based methods (MRI/CT) [6], topology optimization [7], and methods for the optimization of scaffolds microstructure geometry based on mechanobiological criteria [8].

Among the different methods, implicit surface modeling (ISM) is potentially advantageous, since it offers the capability to develop architectures using a single mathematical equation, thus allowing obtaining a compact representation of potentially complex surfaces [9]. Attractive candidates for the design of biomorphic scaffold architectures through implicit functions belong to the large class of triply periodic minimal surfaces (TPMS) [10]. TPMS are, mathematically, defined as surfaces with zero mean curvature everywhere over the entire surface and periodic in three directions extending infinitely. They are naturally occurring in nature and examples include some biological structures, block copolymers, and equipotential surfaces in crystals [11]. Different surfaces attributable to this class are known and also considered for scaffold design [12–14]. Starting from a TPMS surface, it is possible to build solid architectures both by replicating a single TPMS cell in three orthogonal directions and by combining cells with different geometries in order to obtain graded porosity scaffolds [15, 16]. In any case, the study of a unit cell can give useful information for the use of these surfaces in scaffold design. Among the various TPMS, the Schwarz's Primitive (P) minimal surface (hereinafter referred as P-surface) has been considered for different applications including the development of a new type of cellular materials, called shellular, for supporting loads at very low density [17]. In this paper, the P-surface has been investigated for scaffold design. It has been analyzed in terms of analytical parameters that were varied in order to obtain surfaces with different geometrical configurations. Solid P-cells, which are the unit components of a scaffold, were also developed and considered in this analysis. Significant properties for scaffold applications of the cells were determined in relation to geometry and mechanical performance.

Different are the geometrical properties that can affect the performance of a scaffold. Porosity, pore size, and pore interconnectivity are among the main cell properties to be taken into account in the design of the architecture. High values of porosity are generally required, since it improves the conditions for cell ingrowth and nutrient transformation. Significantly enhanced cell proliferation under both static and flow perfusion culture conditions was demonstrated [18] for scaffolds with porosity of 75%, and larger values were suggested [19, 20] to improve cell proliferation. Studies on the influence of pore size on bone ingrowth are also reported in literature and, even if this topic is still under investigation, in some researches pore size values higher than 300 *μ*m were suggested for enhanced cell proliferation [21, 22]. In [23] AM manufactured porous Ti scaffolds with pore size, respectively, of 300 *μ*m, 600 *μ*m, and 900 *μ*m were implanted into rabbit tibia, and it was found that 600 *μ*m and 900 *μ*m scaffolds demonstrated significantly higher bone ingrowth than those with the lowest value of porosity.

Porosity, interconnectivity of the pores, and pore size are correlated to surface area per unit volume, whose high values are beneficial in respect to allowing large numbers of cells to attach to and migrate into porous scaffolds [24], since it influences permeability [25]. Besides surface area per unit volume, curvature also plays an important role in scaffold design [26] and both these geometrical characteristics have also been investigated in the study reported in this paper.

In particular, with regard to the curvature, a correlation between this parameter and tissue regeneration has been shown elsewhere [27], pointing out that concave surfaces are preferable to convex and flat surfaces. Surface curvature that is a local geometrical-differential property of the surface plays, therefore, an important biofunctional role, as confirmed recently by other studies. In particular, the authors of [28] showed that the diameter of 3D spherical pores in scaffold structures affects cell morphology and osteogenic differentiation of mesenchymal stem cells. Moreover, as pointed out in [29], 3D concave substrates promote faster cell migration, while convex substrates induce osteogenic differentiation. This remarkable effect on cellular behavior suggests the importance of the availability of scaffolds with spatially controlled surface curvature in order to optimize tissue regeneration.

The cell properties of a scaffold, especially porosity, can affect the mechanical performance of the implant. Specifically, the stiffness of the scaffold has to be similar to that of the surrounding bone in order to help prevent stress shielding, which can affect the longevity of the implant. High values of porosity and large pores size, while promoting cell ingrowth and nutrient transformation, can lead to a stiffness reduction. Thus, when designing these structures, a compromise is needed between biological and mechanical requirements, which involve the choice of adequate materials and the knowledge of the mechanical properties in relation to the geometry of the cell considered. As for the material, biocompatibility is a basic property to be taken into account, and both biodegradable polymeric materials and metallic alloys are available for these applications [30]. The stiffness evaluation of solid P-cells has been considered in this study by a numerical analysis with finite elements method (FEM) performed on representative solid cells with different geometrical configurations. Titanium alloy (Ti-6Al-4V) made solid cells were considered and subjected to compression load and to shear load to evaluate their stiffness for different values of some key parameters affecting the cell geometries and properties. The effects of some geometrical variations, such as surface thickness and surface radius, on porosity and mechanical properties of two different types of TPMS cells, including the P-surface, were considered in [31]; however, a detailed analysis on the several factors involved in the design of the scaffolds has not been addressed. The aim of this study is to provide a comprehensive characterization of the P-surface for its use in scaffold design, including its application in the design of structures with gradient architecture that can be obtained with a combination of unit cells with different geometries.

This paper has the following outline.  Section 2 discusses the effect of the analytical parameters modulation on P-cell architecture.  Section 3 defines the set of cell properties considered and investigates on the effect of parameters variation on those. This set includes also surface curvature, given the important role it has in cellular proliferation and differentiation.  Section 4 focuses on the design process of a solid P-cell, highlighting the various phases and the design inputs. Finally, in Section 5 a FEM evaluation of the structural behavior of the solid P-cells is carried out. Conclusions are drawn in Section 6.

## 2. Geometric Modeling of TPMS

Several are the approaches generally used for geometric modeling of minimal surfaces. These can be classified into* parametric*,* implicit*, and* boundary *method. In the parametric approach, a function maps a region of a plane to a region of the surface. However, only a few minimal surfaces are simply parameterized and defined by analytic functions in R^3^. Some of these are the catenoid, the helicoid, and the two-dimensional periodic Scherk surface. The infinite minimal surfaces that are periodic in the three dimensions, such as the TPMS, cannot be defined by analytical functions in the Cartesian space. Another possibility of generating them parametrically is to use the Enneper-Weierstrass representation [32]. This parameterization is based on the Weierstrass function that is known, however, only for some minimal surfaces. It can actually be built if there is a surface patch from which the surface can be generated by reflection or rotation around the patch border, but not all the surfaces satisfy this property [33, 34].

The boundary method is based on the iterative refinement of an initial polygonal model defined by its boundary, subject to a variety of boundary and interpolygonal constraints. There are several approaches for generating minimal discrete surfaces using the boundary method, and a critical review of these approaches can be found in [35]. Among them, there is the Plateau method [36] that starts from a description of the boundary of a surface, usually in the form of a polygon in R^3^. The surface is then iteratively refined to minimize the area of the polygonal mesh spanning the boundary. The spanning surface is iteratively refined until no single vertex of the triangulation can be moved further to decrease its area. Based on the principles of this approach, Brakke [37] developed Surface Evolver, a general-purpose application that minimizes the energy of a polygonal mesh subject to constraints through periodic translation and reflection of the evolved patch. In [38] the authors explored TPMS under a constraint in the volume fraction of the phases that the surface separates using the level set method. This constraint is also at the basis of the phase-field method proposed in [39] for generating a triply periodic surface with a constant nonnull mean curvature.

In the implicit method, the surface is defined by means of an implicit function. An implicit function is a continuous scalar-valued function over the domain R^3^. The implicit surface of such a function is the locus of points at which the function takes the zero value. Positive values are inside the surface defined by the implicit function and outside of it there are negative values. Since any periodic surface can be described by the sum of an infinite number of Fourier terms, the TPMS can be approximated by the periodic nodal surfaces (PNS) based on a finite number of trigonometric functions. The quality of this approximation depends on the number of terms of the Fourier series. In [40] the authors showed that the topology of the TPMS is well approximated by a PNS obtained truncating the Fourier series to the leading term, although this kind of PNS is neither minimal nor constant-mean curvature surface. At present, this representation, providing a more readily accessible, though approximate, description of TPMS, is widely used. In this paper, the PNS representation, implemented using an original MATLAB function, describes the outer surface of the solid cell. In particular, this analytical representation was suitably parameterized in order to investigate the effect of the analytical parameters variation on the geometrical configuration and consequently on several properties of the cell.

The P-surface can be described, to the first order of approximation, by the following nodal equation:

(1) $$\begin{array}{c} f\left(\;x,y,z,s,k\;\right)=\cos\frac{x}{s}+\cos\frac{y}{s}+\cos\frac{z}{s}=k \end{array}$$

under the boundary conditions x = [-*π*, *π*], y = [-*π*, *π*], and z = [-*π*, *π*]. This surface divides the cubic cell of side length *L* equal to 2*π* mm into two distinct phases. Phase 1 and phase 2 are, respectively, the regions where *f*(*x*, *y*, *z*, *s*, *k*) < *k* and *f*(*x*, *y*, *z*, *s*, *k*) > *k*.

The architecture of the P-surface can be changed by varying the two analytical parameters* k* and* s* in (1). For a given couple of *s* and *k* values, the MATLAB function describes the P-surface in the form of a tessellated model, generating as output a .*stl* file. The degree of approximation, with which the discrete model represents the P-surface, is affected by the grid resolution, expressed as the number N of grid points. Increasing this value, the P-surface is better approximated by the mesh so that the cell properties can be accurately estimated from the tessellated model. The N-value has been selected to obtain an optimal balance between the need for limiting the number of triangular facets of the tessellated model and that for describing accurately the P-surface. In order to quantify this last aspect, the value of the surface area of the P-cell has been considered as reference. In the case of a continuous P-surface with *k* = 0 and *s* = 1, this value is equal to 2.3451 [41] and it is well approximated by a discrete model with a grid resolution N equal to 100.

**Figure 1:**
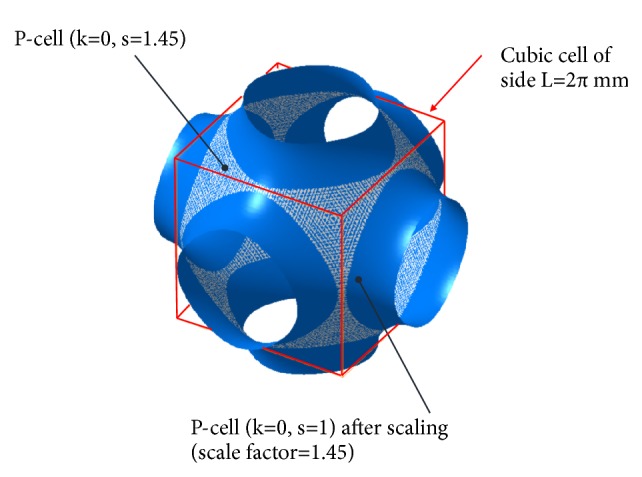
How the modification of the s parameter of the nodal equation (1) affects the P-surface architecture.

The range of variability of *k* and *s* is established by the necessity to preserve the integrity of the P-surface and that of its lateral openings. This requirement is essential to allow the cells to populate gradually and progressively the ducts of the scaffold, regenerating the bone tissues. The* s* modification in (1) has the same effect of scaling the P-cell uniformly with respect to its barycentre, by a scaling factor equal to *s* and truncating it with a cubic cell of side *L* equal to 2*π* mm.  Figure 1 shows, in white dots, the P-cell obtained directly by (1) setting *k* = 0 and *s* = 1.45. This cell lays on the larger blue surface resulting from the uniform scaling with *s* = 1.45 (with respect to the barycentre) of the P-cell with *k* = 0 and *s* = 1 (hereinafter referred to as standard P-cell).

**Figure 2:**
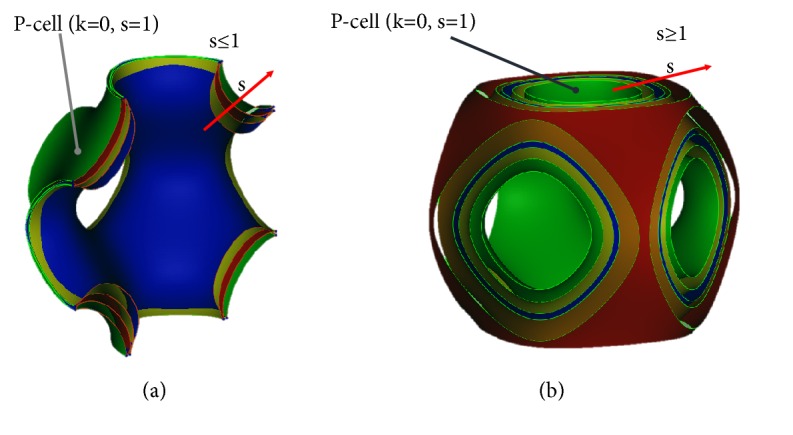
Several P-cells (*k* = 0) obtained varying the* s* parameter.

Figures 2(a) and 2(b) show several models of P-cell (*k* = 0), obtained varying the* s* parameter. In order to preserve the P-cell integrity with *k* = 0, the *s*-parameter must vary within the interval [0.75, 1.5]. In Figure 2(a) the P-cells, shown in middle section to point out better the geometrical differences among the cells, are obtained varying *s* between 1 and 0.75. The value *s* = 1 occurs at the outermost surface (green in the Figure 2(a)). In Figure 2(b) the surfaces are represented for *s*-values higher than 1 and the inner surface (always in green) is obtained for *s* = 1. For* s* values higher than 1.5 the P-cell integrity is not preserved. In both figures, the red arrow shows that* s* values increase going outward.

In this respect, it is important to note that, if a P-cell with* s* parameter other than 1 is replicated along three mutually perpendicular directions, the scaffold generated will be characterized by a nondifferentiable surface, since the continuity C^1^ is not verified at the shared boundary curve between adjacent cells. In [42] the authors observed that there is a contact angle between the P-cells other than 180° when *s* is different from 1 and this value decreases when *s* increases, although remaining still superior to the sharp ninety degree turns in cubic labyrinths.

**Figure 3:**
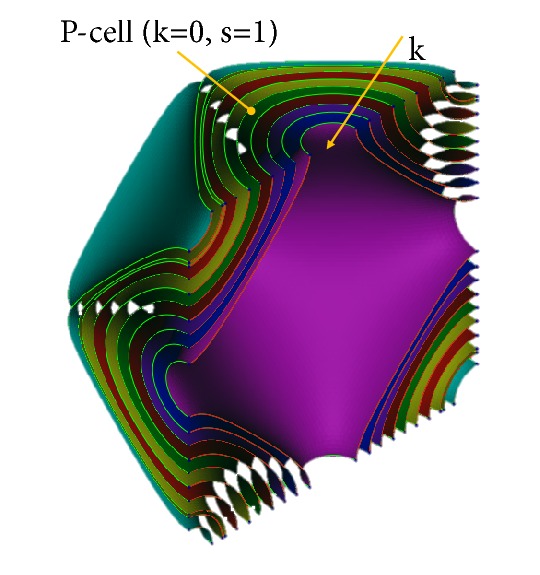
Several P-cells (s = 1) obtained varying the k parameter.

The k modulation allows extracting different level surfaces from the implicit function described by (1). In the case of *s* = 1, the *k* parameter must vary in the interval ]-1,1[: the P-cell integrity, in fact, is not preserved for higher values and its lateral openings are closed for lower values. Several P-cells are represented in Figure 3 for* s*=1 and with* k* parameter varying between -1 and 1. The* k* values increase going inward.

## 3. P-Cell Properties Evaluation

Cell properties are important functional requirements in the design of customized bone scaffolds as they can significantly influence scaffold performance, in terms of both tissue regeneration mechanisms and mechanical behavior. Since the modulation of* s* and* k* in (1) involves, as shown, the modification of the P-surface architecture, the effect of this modulation on the cell properties is investigated below.

Given the important role of the surface curvature in cellular migration and differentiation mechanisms, a new class of properties is proposed here, in addition to the global properties, such as volume fraction and pore size. These new properties aim at quantifying both the curvature and the area of some cell local regions, concave or convex, that are important for tissue regeneration mechanisms.

**Figure 4:**
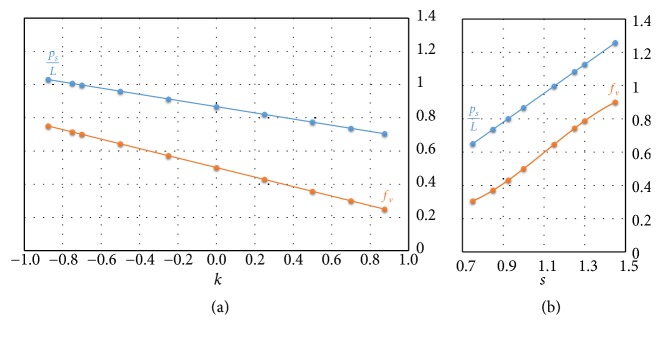
Trend of volume fraction *f*_*v*_ and of pore size *p*_*s*_/*L* with (a) *s* = 1 and *k*∈] − 1  1[ and (b) *k* = 0 and *s* ∈ [0.75  1.5[.

Among the global properties, important functional requirements for the scaffold design are volume fraction *f*_*v*_ and pore size *p*_*s*_. Volume fraction *f*_*v*_ is the cell property defined by the following ratio:

(2) $$\begin{array}{c} f_{v}=\frac{V_{P-\text{surface}}}{V_{\text{unitcubiccell}}} \end{array}$$

where *V*_*P*−*surface*_ is the internal volume delimited from the P-surface and *V*_unitcubiccell_ is the volume of the cubic cell or equivalently the volume of its bounding box. In Figure 4(a)*f*_*v*_ is represented varying *k* for a P-cell with *s* = 1; in Figure 4(b)* k*=0 and* s* varies within the corresponding range of variability. When *k* = 0 and *s* = 1, the P-surface delimits, within the cubic cell, two phases with the same volume (*V*_*P*−surface_ = *V*_unitcubiccell_/2), so *f*_*v*_ is equal to 0.5.

As shown by the diagrams of Figures 4(a) and 4(b), the modification of both *k* and *s* determines a volume fraction variation: *f*_*v*_, in particular, decreases linearly as *k* increases and rises, in a quite linear way, when* s *increases.

**Figure 5:**
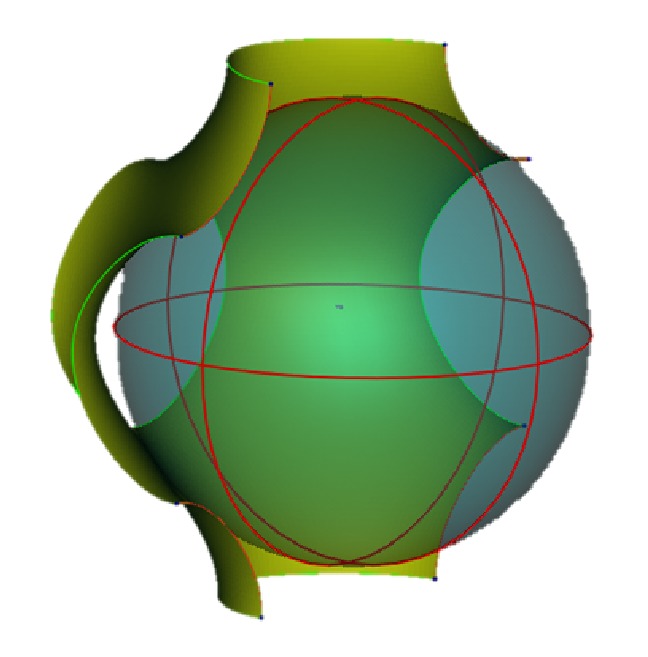
Pore size definition.

Pore size (*p*_*s*_) is the cell property evaluated as the radius of the maximum sphere inscribable in the cell pore, as shown in Figure 5.

In Figure 4(a) the trend of *p*_*s*_ as a function of *k* is represented for a P-cell with *s* = 1; in Figure 4(b)*k* = 0 and *s* varies between 0.75 and 1.5. In order to make the pore size trend independent of the cell size, Figures 4(a) and 4(b) report the dimensionless ratio between the pore size and the length *L* of the cubic cell side on the y-axis of the two diagrams. As shown by the two diagrams, the pore size, coherently with the volume fraction, decreases linearly when* k* increases and rises linearly when s augments.

**Figure 6:**
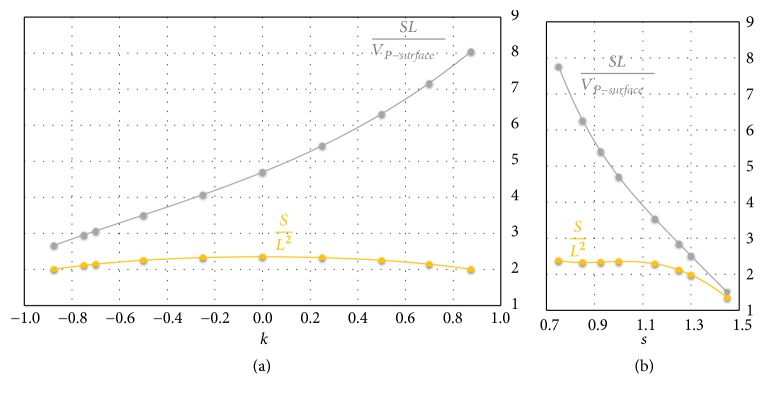
Trend of area-volume and surface ratio for a P-cell with (a) *s* = 1 and *k*∈] − 1  1[ and (b) *k* = 0 and *s* ∈ [0.75  1.5[.

The diagrams of the Figures 6(a) and 6(b) show the trend of two other global cell properties: surface area per unit volume and surface ratio. Both the properties include the area S of the P-surface, which affects significantly the biological processes: high values of S, in fact, facilitate the nutrients and metabolic wastes exchange. Surface area per unit volume is defined as the ratio between S and the internal volume *V*_*P*−surface_ delimited from the surface. This ratio, also called specific surface area, affects significantly, together with pore size, the permeability of a porous material.  Figure 6(a) shows the trend of area-volume ratio varying *k* for a P-cell with *s* = 1; in Figure 6(b)*k* = 1 and *s* varies between 0.75 and 1.5. The ordinate axes of both diagrams report area-volume ratio made dimensionless by side length *L* of the cubic cell. As shown by the two diagrams, surface area per unit volume rises when *k* increases and decreases quickly when* s* augments.

Surface ratio is a cell property defined as the dimensionless ratio *S*/*L*^2^ between the surface area* S* of the P-cell and the area of a square having a side length equal to* L*. This ratio represents an important property of the cell since it enters, as shown later, in the definition of porosity for solid cell. Surface ratio is reported in Figure 6(a) varying* k* for a P-surface with *s* = 1; in Figure 6(b)*k* = 0 and *s* varies in the related range of variability. As shown by Figure 6(a), *S*/*L*^2^ trend is symmetrical with respect to *k*. Surface ratio varies with *k* and *s* and, therefore, with the P-surface architecture, in a limited way. A more relevant dependence on* s* is observed only for values quite larger than 1 (Figure 6(b)).

The analysis of the diagrams in Figures 4 and 6 shows that the global properties of the P-cell are generally a bit more sensitive to the* s *than to* k* modulation, although, as noted above, the shortcoming of generating a nondifferentiable surface must be considered for a scaffold, whose unit cell is characterized by an* s*-value different from 1.

**Figure 7:**
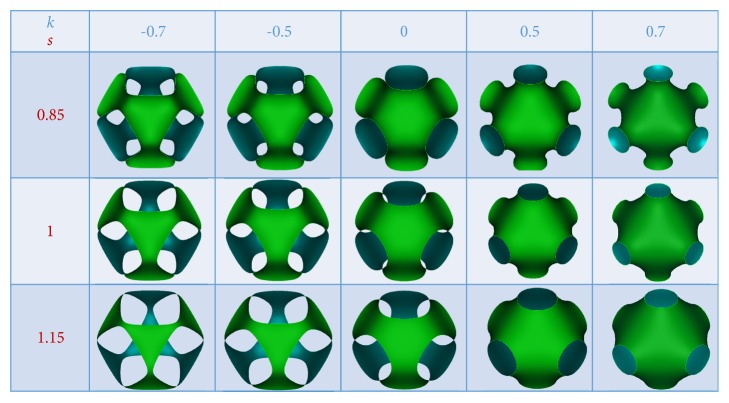
Several geometries of P-cells obtained for different combinations of *k* and *s* parameters.

Cell properties have been evaluated also for other combinations of* s* and* k* parameters. In Figure 7, several P-cells obtained with different combinations of* k* and* s* values are shown.

**Figure 8:**
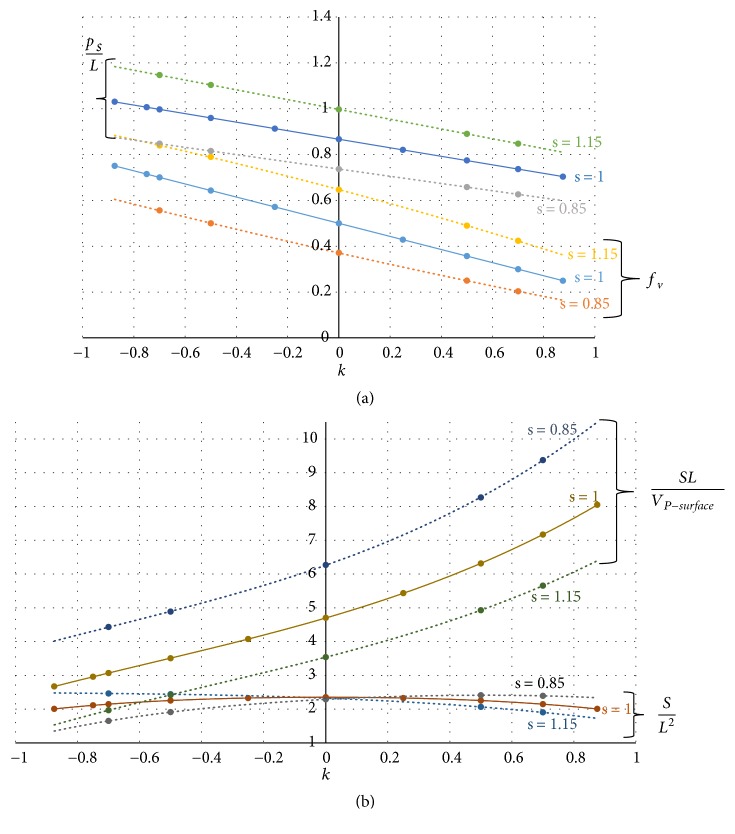
(a) Trend of volume fraction *f*_*v*_ and pore size *p*_*s*_/*L* with varying *k* and for some* s* values. (b) Trend of area-volume ratio and surface ratio with varying *k* and for some* s* values.

Figures 8(a) and 8(b) report the cell global properties *f*_*v*_, *p*_*s*_/*L*, surface area per unit volume, and surface ratio for the P-cells of Figure 7.

### 3.1. Surface Curvature Properties

As already observed, surface curvature is a local property that is important to consider during the design optimization of advanced tissue engineering scaffolds: it contributes, in fact, to the control of the kinetics of tissue deposition and the cellular differentiation during the regenerative processes. The new cell properties, proposed here to take into account these aspects during the scaffold design, require the evaluation of some local geometric-differential properties of the P-surface, such as the principal curvatures.

Let *k*_1_ and *k*_2_ be the principal curvatures at a generic point **p** of the P-surface. The product *k*_1_*k*_2_ of the two principal curvatures defines the Gaussian curvature G, and the average (*k*_1_ + *k*_2_)/2 is the mean curvature H. For a minimal surface *k*_1_ = −*k*_2_ at each point, so that the surface is composed exclusively of saddle points and the curvature H is zero everywhere. However, the P-surface, described by (1) truncating the Fourier series to the leading term, is only approximately a minimal surface when* k*=0; in this case, the surface is locally nearly saddle-shaped and H ≈ 0 at each point. These properties are kept even if the* s* parameter of (1) is modified. In all the other cases (*k* ≠ 0), the P-surface is not minimal and it is characterized by an H-value that is approximately constant at each point of the surface. In this case, in addition to the saddle points, some regions of concave or convex points, according to the sign of the *k*-parameter, appear.

In order to identify these regions, which can affect differently the tissue regeneration, a methodology has been suitably developed here. This methodology, firstly, evaluates the surface curvature maps by the* paraboloid method* and, secondly, recognizes automatically the concave or convex regions estimating, for each of them, how much the surface deviates from being flat, and the corresponding region area. This study has been carried out for the P-surfaces of Figure 7, with the purpose of investigating the relationship with *k* and *s* parameters.

**Figure 9:**
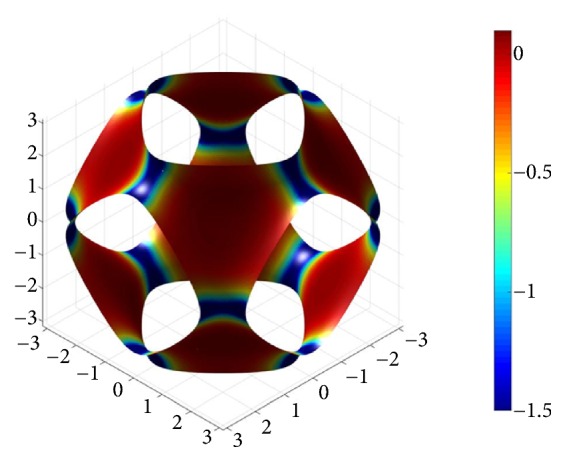
Color map of G distribution for a P-surface with* k* = -0.7 and* s* = 1.

The paraboloid method estimates the curvature by performing a local approximation of the vertices of the triangular mesh with a paraboloid and then deriving the second-order differential properties from that. A MATLAB implementation of this method, developed in [43], has been used here to evaluate the surface maps of the principal curvatures *k*_1_ and *k*_2_.  Figure 9 shows, for example, the surface map of the Gaussian curvature G for a P-surface with* k* = -0.7 and *s* = 1. Nonhomogeneous surface distributions have been obtained for *k*_1_ and *k*_2_ too.

**Figure 10:**
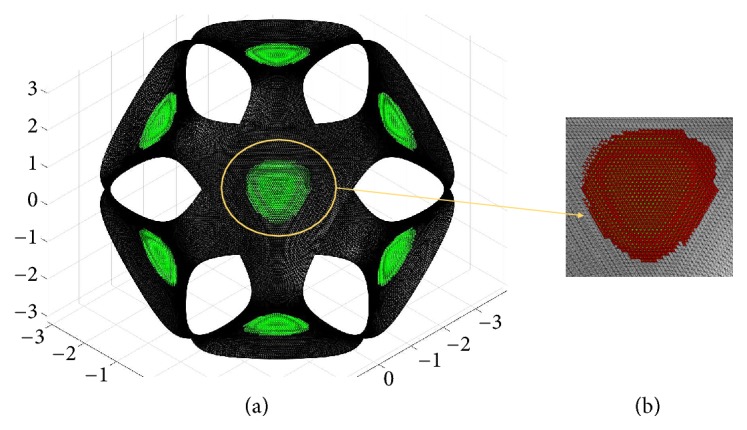
(a) Concave regions recognition for the P-surface with* k* = -0.7 and* s* = 1. (b) A magnified view of one of these regions.

The recognition process of the concave and convex regions from P-surface is based on the sign of the principal curvatures. The algorithm proposed here recognizes a generic node **p** of the mesh as concave (respectively convex) if the principal curvatures *k*_1_ and *k*_2_, evaluated at that vertex, satisfy the condition min⁡{*k*_1_, *k*_2_} ≥ 0 and max⁡{*k*_1_, *k*_2_} > 0 (respectively, max⁡{*k*_1_, *k*_2_} ≤ 0 and min⁡{*k*_1_, *k*_2_} < 0). In Figure 10(a) the mesh nodes, recognized as concave, are represented with green dots in the case of a P-surface obtained setting* k* = -0.7 and* s* = 1 in (1).

The next step of the methodology detects the concave (or convex) triangular regions by a region-growing process, implemented into MATLAB. For the P-surface of Figure 10(a), for example, eight concave regions have been identified, practically identical to each other in terms of area and curvature properties. In Figure 10(b) one of these regions has been represented in a magnified view. Several tests, carried out on P-cells obtained by modulating the *k*-value, have shown that concave and convex regions are, respectively, detected for *k* < 0 and *k* > 0. Due to the surface cubic symmetry, these regions are eight and they are practically identical to each other.

**Figure 11:**
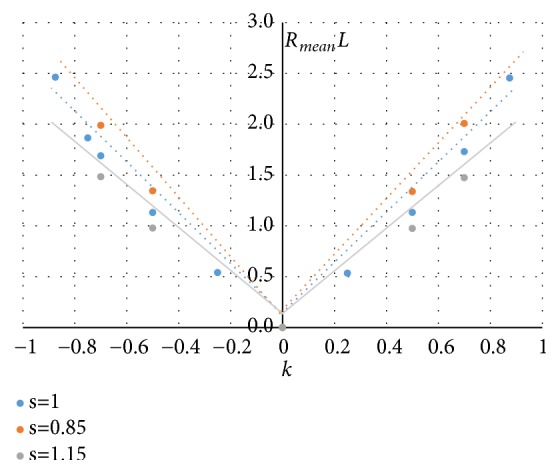
Trend of the mean value of R when* k* and* s* vary.

**Figure 12:**
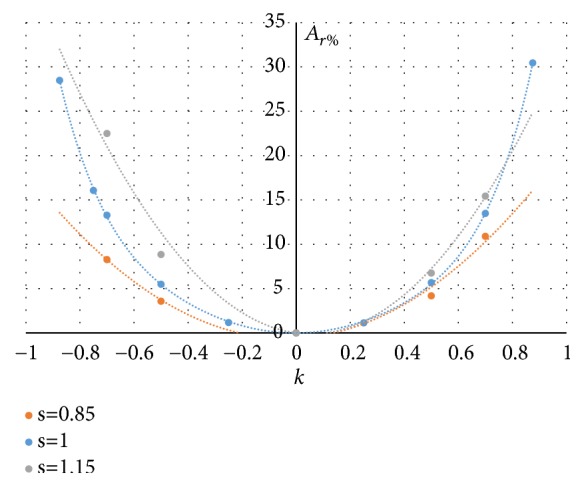
Trend of the *A*_*r*%_ when* k* and* s* vary.

Since the principal curvatures have the same sign at each node of these regions, the curvedness measure *R*, defined as $R=\sqrt{k_{1}^{2}+k_{2}^{2}}$, can be used to measure how much the surface locally deviates from being planar. In Figures 11 and 12 the trend of the mean value of R on all the nodes of a region (in figure the dimensionless product *R*_*mean*_*L*) and the region area *A*_*r*%_ (evaluated as percentage of the total area S of the P-surface) are reported when* k* and* s* vary.

The diagrams of Figures 11 and 12 show that both the curvedness *R*_*mean*_ and the area *A*_*r*%_ depend significantly on *k*, increasing with the absolute value |*k*|. These two local properties are also affected by the* s parameter*: for a given* k*-value, the curvature *R*_*mean*_ increases and the area *A*_*r*%_ decreases when* s *reduces.

## 4. Solid P-Cell Design

**Figure 13:**
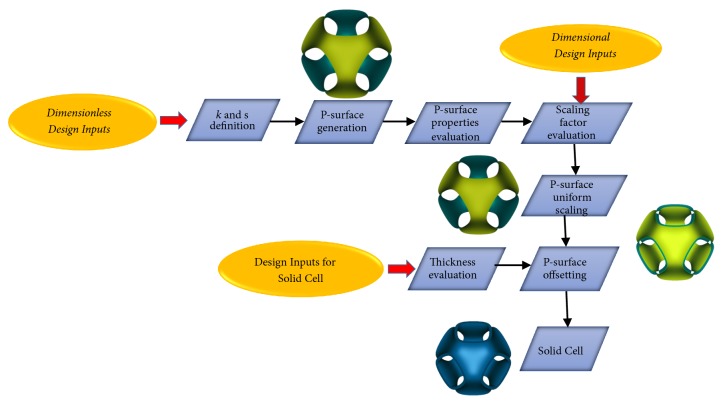
Flowchart of the design process of a solid P-cell.

A flowchart of the design process of a solid cell, starting from an external P-surface that meets various design requirements, among the cell properties described in the previous section, is shown in Figure 13. These target requirements may be given in the form of dimensionless design inputs (such as volume fraction *f*_*v*_) or not (such as pore size *p*_*s*_ or curvature *R*_*mean*_). The target values of dimensionless design inputs are achieved by selecting appropriately the values to be assigned to the* k* and* s *parameters according to the diagrams for P-surface cell properties shown in the previous section.

In the case of design inputs dependent on the size *L* of the cubic cell (differently, based on the type of the input), an appropriate scale factor, by which a uniform scaling transformation of the P-surface with respect to the cell's centre is performed, has to be identified.

In the flowchart of Figure 13, the first blocks aim at defining the P-surface architecture. This phase requires the identification of the* k* and* s* values to be considered in (1). Then the P-surface is scaled in order to meet the target values assigned for dimensional design inputs. In this respect, it is interesting, for example, to observe that, in order to obtain values of curvature radius able to influence the tissue regeneration (i.e., between 250 and 750 *μ*m, as evidenced in [28]), the final size of the P-cell may be so small that its manufacturing for AM would become quite problematic. This means that the radii of curvature estimated at the points of the concave and convex regions (detected if* k* is nonzero) are therefore not sufficiently high to influence the mechanisms of cellular regeneration. For this reason, the authors intend to apply shortly the methodology, proposed here for the concave or convex region recognition, to other TPMS surfaces characterized by surface curvature maps with higher average values (such as Double Diamond or Fischer-Koch S surfaces).

The next phase of the methodology aims at generating the solid P-cell in the form of a thin shell. This phase requires the definition of another fundamental design variable: the thickness* t*. The* t-*value may be identified, for example, starting from the porosity* P* of the solid P-cell. Porosity, as already observed, is an important target property of the bone scaffold from both biological and mechanical point of view and it is defined by the following formula:

(3) $$\begin{array}{c} P=\frac{{V_{\text{unitcubiccell}}-V}_{P-\text{cell}}}{V_{\text{unitcubiccell}}} \end{array}$$

where*V*_*P*−cell_ is the volume occupied by the solid P-cell.

If porosity *P* is an assigned design input, the thickness *t* can be evaluated from the following equation:

(4) $$\begin{array}{c} P=1-\left(\;\frac{S}{L^{2}}\;\right)\cdot \left(\;\frac{t}{L}\;\right) \end{array}$$

since the dimensionless ratio *S*/*L*^2^ and the L-value were already identified during the previous steps of the flowchart. Once the t-value was established, the solid P-cell is finally obtained from the tessellated surface by an inward offset operation (with an offset distance equal to t). Then, the surface is converted into a solid structure. These last two steps were performed into a CAD environment by CATIA software.

**Figure 14:**
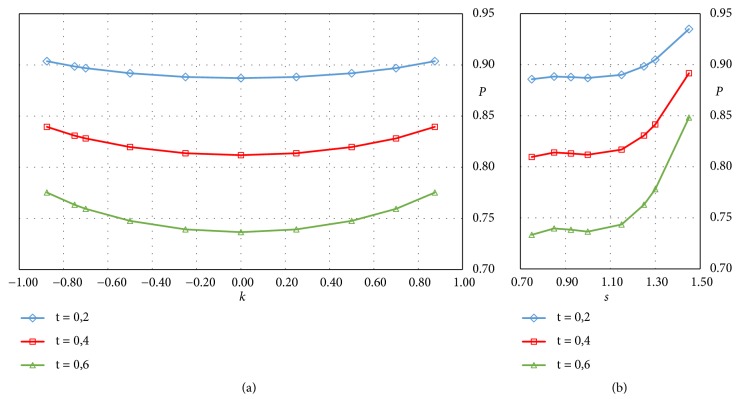
Trend of the porosity* P*, evaluated the formula (3), for solid P-cells: (a) in the case of* s* parameter variable (*k* = 0) and (b)* k* variable (*s* = 1).

Figure 14 reports the trend of porosity* P*, estimated from the volume occupied by the solid cell, for several cells obtained by modulating the two parameters* s *and* k*.

Three different values of the thickness* t* have been considered, and each curve in the diagrams of Figure 14 is relative to a constant value of the* t*-parameter, expressed in mm. In accordance with the trend of the surface ratio *S*/*L*^2^, shown in Figure 6, the porosity is little affected by the* k* and* s* modulation, for* s* values lower than 1.1 and for a given value of the thickness. The effect of this variation, however, is more relevant at high thickness values than low ones. The thickness variation, on the other hand, has a more relevant effect, which, as expected by (4), is linear with* t*.

## 5. Structural Numerical Evaluation of the Solid P-Cells

**Figure 15:**
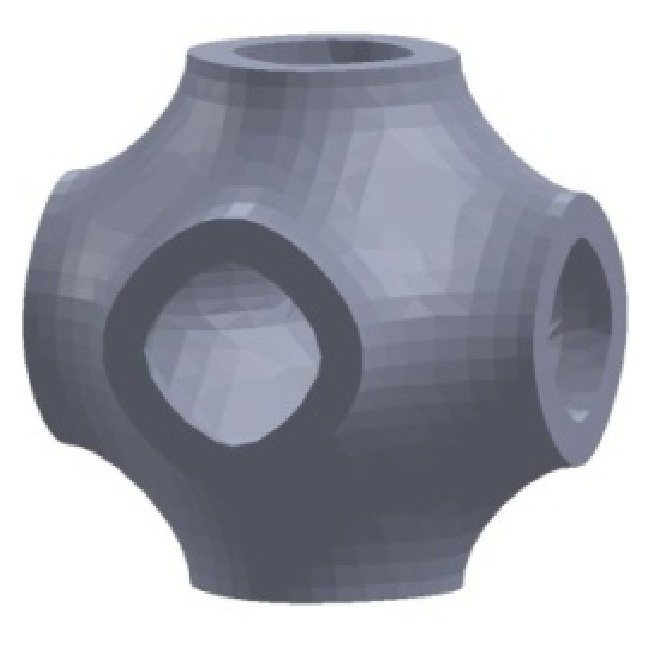
Solid P-cell (*k* = 0, *s* = 1).

The introduction of the thickness on the P-cell, as previously described, allows obtaining a solid P-cell as that reported in Figure 15 and derived from the standard P-surface (*k* = 0, *s* = 1).

The geometrical configuration of a solid P-cell can be changed by the variation of the key parameters and the resultant architecture can affect the stiffness of the cells, which is an important issue in the design of the scaffolds.

**Figure 16:**
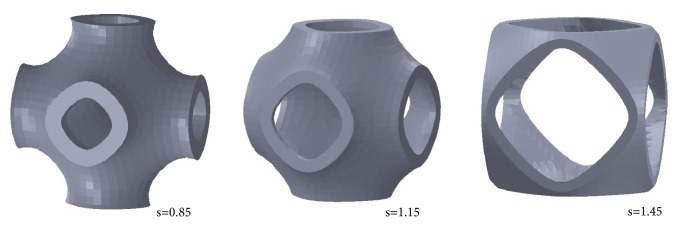
Architectures of solid P-cells with different values of the parameter* s* and *k* = 0.

In order to analyze the behavior of the solid P-cells, representative configurations can be selected, given that the cells can be structurally characterized by analyzing a limited number of them. In particular, solid P-cells were derived by varying the* s* parameter (*k* = 0).  Figure 16 shows some of the cells considered.

**Figure 17:**
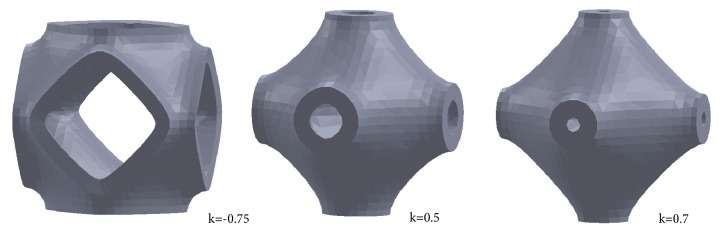
Architectures of solid P-cells with different values of the parameter *k* and *s* = 1.

The figure reports the solid P-cells obtained, respectively, for *s* = 0.85, *s* = 1.15, and *s* = 1.45. A qualitative comparison among the solid P-cells highlights, at the same thickness, the different geometrical configurations. Similarly, a discrete number of solid P-cells with the *k* parameter variable (*s* = 1) were considered.  Figure 17 shows some representative geometrical configurations of these solid P-cells.

The figure reports the solid P-cells, respectively, obtained for* k* = -0.75,* k* = 0.5, and k = 0.7. The differences between the resultant architectures, at the same thickness, are qualitatively highlighted also in comparison with the solid P-cells reported in the previous Figure 16.

The models were structurally analyzed by means of finite elements analysis. The biocompatible material chosen for the solid P-cells was a titanium alloy (Ti–6Al–4V), which is considered advantageous for these applications thanks to its excellent osseointegration, superior corrosion resistance, and good mechanical properties [44]. The properties of the constituent material were assumed as follows: elastic modulus E_s_ = 110GPa, shear modulus G_s_ = 40GPa, and Poisson's ratio *ν*_s_ = 0.3. Solid P-cells with a size of 5mm were considered. Each solid model was imported into a FE code and meshed by using eight nodes linear brick elements. Convergence testing was performed in order to minimize the influence of mesh density on the results. A global element size of 0.05 was chosen as an optimal compromise between mesh sensitivity and computational effort required.

**Figure 18:**
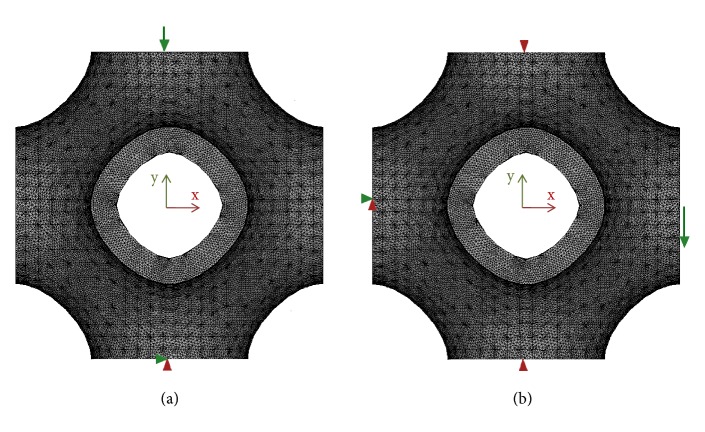
Loading configurations considered: (a) uniaxial compression load; (b) shear load.

Two different loading configurations were considered. In particular, each model was subjected to compression load and to shear load in order to determine the elastic moduli for the two loading configurations.  Figure 18 reports, schematically, the boundary and loading conditions considered for the models of the solid P-cells.

Uniaxial compression tests were simulated by applying a uniform displacement, within the material elastic limit, to the top surface of the solid P-cell in the y direction (Figure 18(a)), corresponding to 0.1% of a compressive strain, while the lower surface of the model was fully constrained.

The elastic modulus E, given by the ratio between the homogenised stress and the applied strain, can be calculated by means of the relationship *E* = (*F*_*R*_/*A*)/*ϵ*_*A*_, where *F*_*R*_ is the reaction force calculated by the FE solver, A is the total cross-sectional area, and *ϵ*_*A*_ is the applied strain. For each solid P-cell three different values of the thickness were considered in order to verify the influence of this parameter on the stiffness of the cell.

**Figure 19:**
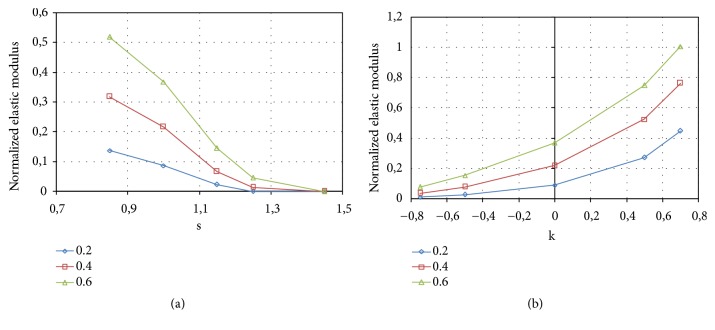
Normalized elastic modulus: (a) solid P-cells with variable* s* parameter (*k* = 0); (b) solid P-cells with variable* k* parameter (*s* = 1).

The results obtained for the analyzed solid P-cells are reported in Figure 19. In particular, Figure 19(a) shows the normalized elastic modulus (E/E_s_) relative to the solid P-cells with the* s* parameter variable (*k* = 0), while Figure 19(b) reports E/E_s_ for the solid P-cells with variable* k* parameter (*s* = 1).

Each curve in the diagrams of Figure 19 corresponds to a constant value of the thickness, reported in each graph and expressed in mm. As expected, at constant *s* or *k*, an increase of the stiffness of the solid P-cell with the increasing of the thickness can be observed.

From the diagram in Figure 19(a) it is possible to observe that the values of the normalized elastic modulus decrease as the parameter *s* increases with an approximately linear trend. Inversely, for the solid P-cells with *k* variable, the elastic modulus grows with the increasing of the value of the parameter* k*, as shown in Figure 19(b), and the intermediate value corresponds to that of the standard solid P-cell (k = 0). From a comparison between the values obtained for the two types of solid P-cells, it can be observed that, for any given thickness, on average, higher values of the elastic modulus are obtained for the solid P-cells with *k* variable with respect to those where* s* is the parameter that is varied.

**Figure 20:**
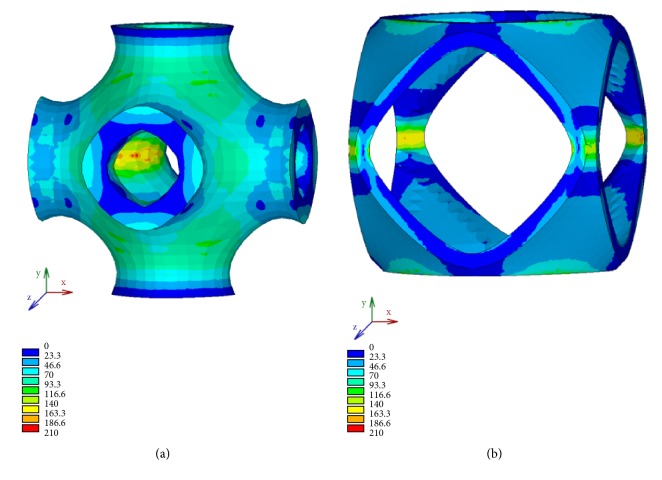
Von Mises stress distribution for different values of the parameter s: (a) *s* = 0.85; (b) *s* = 1.45.

FE simulations allow mapping the stress distribution in the solid P-cells that, in this analysis, can be useful to compare, in terms of strength, the geometries of the cells considered.  Figure 20 shows the isocolor representation of Von Mises stress, expressed in MPa and obtained for different values of the parameter s. The maps are relative to the intermediate value of the thickness.

The solid P-cell reported in Figure 20(a), corresponding to *s* = 0.85, falls in the range of the cells with the highest values of stiffness, while the cell in Figure 20(b) corresponds to a model (*s* = 1.45) with a low value of the elastic modulus. Generally, a structure with higher average stress at a constant applied strain has higher elastic properties. This is also verified in this case by a qualitative comparison between the maps reported in Figure 20 and the corresponding values of the compressive elastic modulus. In fact, the solid P-cell *s* = 1.45 shows on average lower stress values with respect to the other considered.

**Figure 21:**
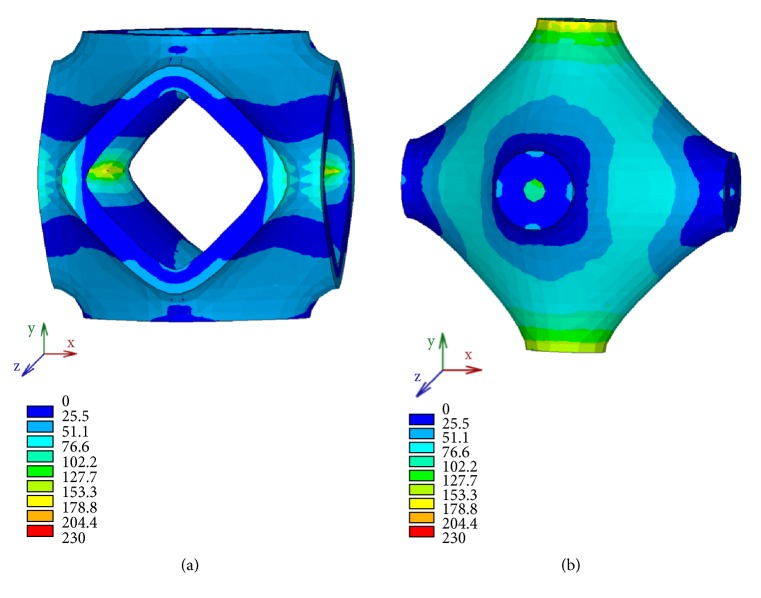
Von Mises stress distribution for different values of the parameter *k*: (a) *k* < 0; (b) *k* > 0.

Similar considerations may apply to the solid P-cells with* k* as variable parameter. Von Mises stress distribution, expressed in MPa, of two representative solid P-cells belonging to this class is reported in Figure 21. The maps are relative to the same value of the thickness previously considered for the cells with s variable.

In particular, Figure 21 shows the isocolor representation of Von Mises stress, respectively, for a negative value of the parameter, namely,* k* = -0.75 (Figure 21(a)) and a positive value (*k* = 0.7) reported in Figure 21(b). The lower average stress in the solid P-cell with* k* = -0.7, characterized by a low value of the elastic modulus, is highlighted with respect to the other solid P-cell reported in the figure which belongs to the group of cells having high values of stiffness. A qualitative analogy between the maps reported, respectively, in Figure 20 and in Figure 21 can also be observed.

**Figure 22:**
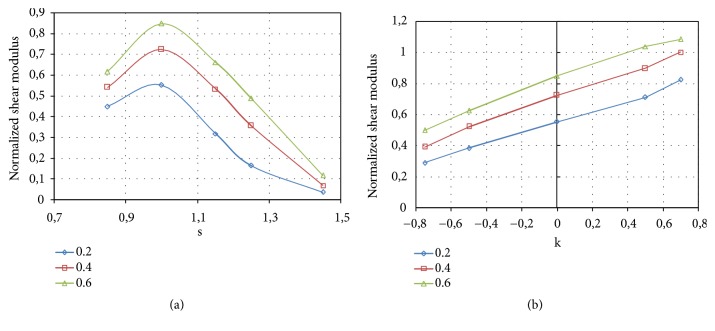
Normalized shear modulus: (a) solid P-cells with variable s parameter (*k* = 0); (b) solid P-cells with variable *k* parameter (*s* = 1).

The shear modulus was evaluated by the application, to each model of the solid P-cells, of the loading configuration schematically reported in (Figure 18(b)). A uniform displacement was applied to the nodes on the outermost lateral face (+x) in the y direction, while the opposite face (-x) was fully constrained. The nodes on the top face (+y) of each model and those on the bottom face were also constrained in the x direction. The shear modulus G, analogously to E, was evaluated starting from the reaction force calculated by the FE solver. The equivalent applied strain was 0.1%.  Figure 22 reports the results of the FE simulations relative to shear tests. The figure depicts the normalized shear modulus (G/G_s_) obtained for the solid P-cells for different values of the* s* parameter (Figure 22(a)) and that relative to the cells with variable k parameter (Figure 22(b)). Each curve corresponds to a constant thickness, reported in each graph and expressed in mm.

From Figure 22(a) it can be inferred that the shear modulus has approximately a parabolic trend, with the maximum value corresponding to the standard solid P-cell characterized by a unitary value of the parameter s. The shear modulus relative to the solid P-cells with variable* k* parameter, reported in Figure 22(b), has an increasing and nearly linear trend analogously to the compressive modulus. From the diagram it is also possible to observe that, in correspondence of higher values of the parameter *k* and at the highest thickness considered, the cells have a shear modulus higher than that of the bulk material, which implies that the shear performance can be increased above the reference. As regards Von Mises stresses, observations similar to that exposed for the solid P-cells subjected to compression load, previously considered, are applicable since similar results were obtained.

**Figure 23:**
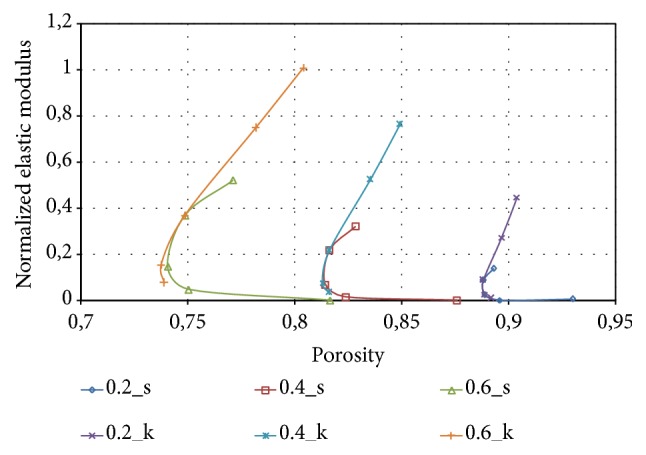
Normalized elastic modulus as a function of porosity.

The relationship between stiffness of the solid P-cells, expressed in terms of elastic moduli, and porosity has also been considered since it is an important issue for the design of the scaffolds. The calculated elastic and shear moduli can be expressed as a function of porosity, which is related to the relevant geometric parameter S/L^2^, discussed in the previous paragraphs, by means of (4).  Figure 23 reports the normalized elastic modulus obtained for the two classes of the solid P-cells analyzed in this study, labelled in the figure as* s* and* k*, at the different values of thickness considered.

Figure 23 shows that the solid P-cells obtained by combining the different parameters are characterized by stiffness values that can be varied within a quite wide range maintaining at the same time high values of porosity, which is a requisite characteristic in scaffold design. In fact, high porosity is recommended since it is a critical parameter in promoting vascularization and tissue ingrowth. From the diagram it is also possible to observe that, at a constant thickness, a nearly parabolic curve is obtained whose upper portion is made with the values of the elastic modulus of the cells with variable *k* parameter, while the lower portion identifies the solid P-cells with variable* s* parameter.

**Figure 24:**
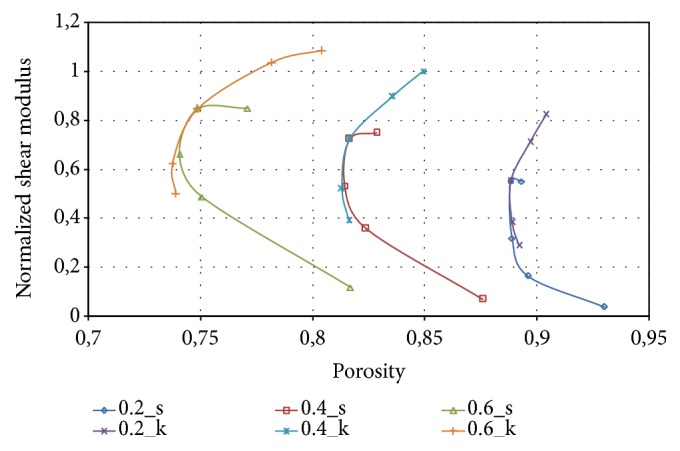
Normalized shear modulus as a function of porosity.

The shear modulus, obtained for the different solid P-cells, has also been related to porosity.  Figure 24 reports the variation of the normalized shear modulus as a function of this parameter for both types of solid P-cells considered, respectively, labelled with *s* and *k*.

Similarly to the compression modulus, each curve obtained at constant thickness has a nearly parabolic trend and globally different shear moduli are obtained in a range of values of porosity suitable for scaffold design.

## 6. Conclusions

In this paper, a comprehensive characterization of the P-cell for its application in design of bone scaffolds was carried out. Several cell properties, able to affect the performance of a scaffold in terms of tissue regeneration and mechanical behavior, were assessed for various P-cell architectures obtained by modifying the two analytical parameters of the implicit function used for describing the Schwartz Primitive Surface. These cell properties include, in addition to global properties, also curvature, given the important role it has in cell proliferation and differentiation.

The evaluation of the cell global properties from several P-cell architectures shows a bit higher sensitivity to the modulation of* s* than to that of* k*–parameter. The repetition of a cell with* s* different from 1 along three mutually perpendicular directions, however, leads to defining a scaffold, whose C^1^ continuity is not verified at the shared boundary curve between adjacent cells. This aspect should be considered especially for* s* values very different from 1.

To quantify surface curvature variation due to* k* and* s* modulation, in this work new local cell properties have been proposed, which are useful to consider when designing scaffolds with spatially controlled surface curvature. In particular, these properties require, to be evaluated, the estimation of the principal curvatures maps locally to some regions potentially critical for cellular regeneration mechanisms. An original methodology has been developed here to detect automatically these regions. The application of this methodology to several P-cells pointed out that the radii of curvature, estimated at the points of the concave and convex regions detected, are not sufficiently high to influence the mechanisms of cellular regeneration and differentiation. The methodology, however, can be used for any other cell, regardless of its geometry. In this respect, the authors intend to apply it to other TPMS surfaces characterized by surface curvature maps with higher average values (such as Double Diamond or Fischer-Koch S surfaces).

The structural numerical analysis on the solid P-cells has highlighted the relationship between the parameters* s* and* k* and the elastic moduli of the cells. Moreover, it was found that the values of the stiffness can be varied within a quite wide range, maintaining, at the same time, high values of porosity, which is a main requirement in the design of the scaffolds.

The results of this investigation are important to identify the values to be assigned to the set of parameters defining its architecture (i.e., the analytical parameters *k* and *s*) and sizes (i.e., *L* and *t*). Since the global and local properties of the P-cell represent important functional requirements in the design of customized bone scaffolds, the study also pointed out how they may be integrated into the workflow of the design process of a solid P-cell, with the view to obtaining scaffolds able to satisfy both biological and mechanical requirements.

## Data Availability

The data used to support the findings of this study are available from the authors upon request.
